# Demographic Profile, Etiology, and Perinatal Outcome Associated With Preterm Birth in a Tertiary Hospital of Eastern India: A Retrospective Study

**DOI:** 10.7759/cureus.26066

**Published:** 2022-06-18

**Authors:** Vandana Mohapatra, Sujata Saraogi, Sujata Misra

**Affiliations:** 1 Department of Obstetrics and Gynaecology, Fakir Mohan Medical College & Hospital, Balasore, IND; 2 Department of Paediatrics, Fakir Mohan Medical College & Hospital, Balasore, IND

**Keywords:** retrospective study, spontaneous, risk factors, preterm birth, perinatal outcome, iatrogenic, etiology, demography

## Abstract

Background

Preterm birth (PB), defined as birth occurring at less than 37 weeks of gestation, is a leading cause of perinatal mortality and morbidity in the world.

Objectives

This study aimed to evaluate the socio-demographic characteristics and etiological factors associated with preterm birth and consequent adverse perinatal outcomes retrospectively at a tertiary care hospital.

Methods

A single-centre retrospective observational study was conducted in the department of Obstetrics & Gynaecology, Fakir Mohan Medical College & Hospital, Balasore, Odisha, India, from April 2019 to March 2020. Data were retrieved from the antenatal ward admission register, case files, theatre records, and neonatal care unit records and reviewed. Descriptive statistics were used to describe data. Chi-square test and student’s t-test were used to find significance of difference between variables.

Results

The incidence of preterm birth in the study population was 5.52%. The mean gestational age of preterm deliveries was 34.39 ± 1.92 weeks. The bulk of the women hailed from a rural background and belonged to the lower socioeconomic strata. About 47.29% of the women were nulliparous and spontaneous preterm birth was noted in 70.40%. Premature rupture of membranes (PROM), anaemia, intrauterine growth restriction (IUGR), preeclampsia, and eclampsia were the most common adverse pregnancy conditions prevalent in these women. Preterm deliveries comprised 31.21% of all neonatal intensive care unit (NICU) admissions. Respiratory distress syndrome, birth asphyxia, neonatal sepsis, and jaundice were the most common complications. Neonatal death occurred in 51 (9.21%) preterm infants with birth asphyxia being the commonest cause of such deaths. Maternal factors and adverse neonatal outcome variables were compared between the spontaneous and iatrogenic/medically indicated preterm birth groups. Preeclampsia, IUGR, and cesarean section were more significantly associated with the iatrogenic group.

Conclusion

Our study provides a general overview of the associated etiological factors and perinatal health concerns associated with preterm birth in a rural/semi-urban setting in Eastern India. The findings might provide essential data for taking steps toward the prevention and management of preterm birth from a developing country’s perspective.

## Introduction

Preterm birth, defined as birth occurring at less than 37 weeks of gestation, is the leading cause of perinatal mortality and morbidity in the world [1,2]. With recent advances in prenatal care, the incidence of preterm birth in both the developing as well as the developed world has increased in recent years [3]. Globally, about 15 million preterm deliveries occur annually with a significant disproportionate burden on the developing countries. India is amongst the countries that report the greatest number of preterm births. Asia, along with sub-Saharan Africa, accounts for more than 60% of the world’s preterm babies and over 80% of the world’s neonatal deaths due to complications associated with preterm deliveries [4]. Neonatal mortality increases substantially with decreasing gestational age [5]. Compared with term infants, premature babies are more likely to develop long-term neurological and developmental disorders. Thus, the medical and economic impact of preterm birth is not only appreciated in the perinatal and/or neonatal periods but also extends into adulthood.

The etiology of preterm birth is multifactorial and in the majority, an idiopathic cause is still attributed [6]. There are many maternal or fetal characteristics that have been associated with preterm birth, including maternal demographic characteristics, nutritional status, past obstetric history, present pregnancy characteristics and high-risk factors, infection, uterine contractions, cervical length, and biological and genetic markers [7]. The pathophysiological pathways and biochemical mechanisms leading to the onset of preterm labour are unclear [8]. Moreover, research in this field is lacking in the developing world, which bears the maximum burden. Because of the aforementioned factors, steps to predict, prevent, and manage preterm labour in a particular population are tough in the light of imprecise evidence. Consistent reporting of all pregnancy outcomes associated with this condition in varied clinical settings is crucial to the advance of understanding and monitoring of trends [1]. Identification of risk factors can provide insights into mechanisms of preterm birth and initiation of risk-specific treatment. More accurate data on epidemiology, maternal risk factors, and associated neonatal morbidity and mortality specific to a particular region or setting may enable government health programs to effectively target interventions to reduce preterm birth and improve perinatal outcomes.

This study was, hence, undertaken with the aim to retrospectively evaluate the socio-demographic characteristics and etiological factors associated with preterm births along with the consequent adverse perinatal outcomes at a tertiary care hospital in Eastern India. 

## Materials and methods

A single-centre retrospective observational study was conducted in the department of Obstetrics & Gynaecology, Fakir Mohan Medical College & Hospital, Balasore, Odisha, India, for one year, from April 2019 to March 2020. The procedures followed were in accordance with the ethical standards of the responsible committee on human experimentation (institutional or regional) and with the Helsinki Declaration of 1975, as revised in 2000. The study was approved by the Institutional Ethics Committee of Fakir Mohan Medical College & Hospital, Balasore (approval number 31/IEC, dated August 18, 2020). Consent from the patients was waived off by the ethical committee of the institute because of the retrospective design of the study.

All deliveries between 28+0 and 36+6 weeks of gestation that occurred during the one-year period were included in the study. Only live births were included in the study. We excluded stillbirths, multiple pregnancies, and pregnancies complicated by congenital malformations. Data were retrieved from the antenatal ward admission register, case files, theatre records, and neonatal care unit records and then reviewed. Information sought was socio-demographic characteristics, complete obstetric profile including antenatal maternal high-risk factors, the onset of labour, mode of delivery, and adverse perinatal outcome variables.

The basic socio-demographic variables that we studied were maternal age, residence, socioeconomic class and education. Detailed obstetric profile of the women such as parity, gestational age, previous abortions, prior preterm birth, booking status, and regularity of antenatal care (ANC) visits was recorded. The prevalence of preeclampsia, eclampsia, intrauterine growth restriction (IUGR), antepartum haemorrhage, gestational diabetes mellitus (GDM), prediabetes, anaemia, abnormal fetal heart rate (FHR) pattern, malpresentation, premature rupture of membranes (PROM), oligohydramnios, polyhydramnios, and medical illnesses (fever, urinary tract infection, gastroenteritis) in the study population was noted. The parameter of management considered was the administration of antenatal corticosteroids and magnesium sulphate for neuroprotection. The onset of labour was either spontaneous or iatrogenic and accordingly, the women were grouped into two subtypes. The adverse perinatal outcome variables retrieved were low birth weight, neonatal intensive care unit (NICU) admission, respiratory distress syndrome (RDS), birth asphyxia, neonatal sepsis, neonatal hypoglycemia, hypothermia, jaundice requiring phototherapy, duration of NICU stay, and neonatal death.

Length of gestation was estimated based on the date of the last menstrual period or from the first-trimester ultrasound. Socio-economic status was derived using the modified Kuppuswamy scale [9]. Spontaneous preterm birth was considered when preterm birth occurred as a consequence of spontaneous onset labour pain or premature rupture of membranes. Iatrogenic/medically indicated preterm birth included induced preterm birth for medical indications and maternal or fetal complications, which mandated the termination of pregnancy. Regular ANC comprised four or more antenatal visits; less than four visits were categorized under irregular ANC. Anaemia in pregnancy was defined as haemoglobin concentration ≤ 11gm/dl. Spontaneous rupture of fetal membranes before the onset of labour was taken as PROM in the study. An abnormal FHR pattern was detected by a cardiotocograph. The FHR tracing was classified as normal, suspicious, or abnormal according to National Institute for Health and Care Excellence (NICE) guidelines. Birth weight < 2500 gm was defined as low birth weight. In the study, birth asphyxia was taken as an APGAR score < 7 at the fifth minute. Blood glucose < 40mg/dl was considered neonatal hypoglycemia for the purpose of the study.

All pregnant women symptomatic of coronavirus disease 2019 (COVID-19) or with a history of exposure who were admitted to our facility from February 2020 onward underwent COVID-19 testing as per Government of India and Indian Council of Medical Research (ICMR) guidelines 2020. In this first wave of the pandemic, our protocol was to test those neonates for COVID-19 who were born to positive mothers, had contact history with positive cases, or were symptomatic with pneumonia or severe acute respiratory illness. Testing was done by nasopharyngeal swab reverse transcriptase-polymerase chain reaction (RT-PCR). 

Statistical analysis

Data analysis was done using Microsoft Excel (Microsoft Corporation, Redmond, Washington, United States) and IBM SPSS Statistics for Windows, Version 20.0 (Released 2011; IBM Corp., Armonk, New York, United States). Descriptive statistics were used to describe data. Continuous variables were expressed as the mean and standard deviation, whereas discrete variables were expressed as frequencies and percentages. Association between variables was analyzed using the Chi-square test with or without Yates’ correction and student’s t-test. P-value <0.05 was considered statistically significant.

## Results

During the research period, there were 12,567 total deliveries at our institution, of which 222 were stillbirths and excluded from the study. Of the 12,345 live births, 682 were preterm deliveries. Thus, the incidence of preterm birth in the study population was 5.52%. Among 682 deliveries, 554 met the eligibility criteria of the study after excluding multiple births and pregnancies complicated by congenital malformations. The mean maternal age of women with preterm delivery was 24.27 ± 4.42 years. The socio-demographic profile of women presenting with preterm birth is outlined in Table 1. Most of the women hailed from a rural background and belonged to the lower socioeconomic strata. The obstetric profile is depicted in Table 2. The mean gestational age of preterm deliveries in the study population was 34.39 ± 1.92 weeks.

**Table 1:** Maternal socio-demographic characteristics associated with preterm birth in the study

Variables	Frequency (N=554)	Percentage (%)
Maternal age (in years)		
<20	86	15.52%
20-30	360	64.98%
>30	108	19.49%
Residence		
Urban	119	21.48%
Urban slum	90	16.25%
Rural	345	62.27%
Education		
No formal education	10	1.81%
Primary	123	22.20%
Secondary	250	45.13%
Higher secondary & above	171	30.87%
Socioeconomic class		
Upper class	50	9.03%
Upper middle	56	10.11%
Lower middle	108	19.49%
Upper lower	216	38.99%
Lower	124	22.38%

**Table 2:** Obstetric profiles associated with preterm birth in the study P: parity; ANC: Antenatal care

Variables	Frequency (N=554)	Percentage (%)
Gestational age in weeks		
<34	254	45.85%
34 – 36^+6^	300	54.15%
Gravida		
Primigravida	249	44.95%
Multigravida	305	55.05%
Parity		
P_0_	262	47.29%
P_1_	100	18.05%
P_2_ & above	192	34.66%
Prior preterm birth	63	11.37%
Previous abortions	98	17.69%
Booking status		
Booked	511	92.24%
Unbooked	43	7.76%
ANC visits		
Regular	376	67.87%
Irregular	178	32.13%

The frequency of antepartum complications is shown in Table 3. PROM, anaemia, IUGR, preeclampsia, and eclampsia were the most common adverse pregnancy conditions prevalent in these women. Not a single case of COVID-19 positive mother or neonate was detected during the study period in our institute after testing as per Government of India-ICMR Guidelines. Thus, the COVID-19 pandemic, which is known to adversely affect perinatal outcomes, had no effect on our study. The majority of women (70.22%) in the study population received a complete course of antenatal corticosteroids (Table 4). Injection dexamethasone was administered in all but two cases since the drug is supplied through government sources. The rest two received injection betamethasone. Data regarding the onset of labour and mode of delivery were collected and tabulated.

**Table 3:** Antepartum complications associated with preterm birth in the study IUGR: intrauterine growth restriction; GDM: gestational diabetes mellitus; FHR: fetal heart rate; PROM: premature rupture of membranes; UTI: urinary tract infection

Variables	Frequency (N=554)	Percentage (%)
Preeclampsia/eclampsia	75	13.54%
IUGR	84	15.16%
PROM	166	29.96%
Antepartum hemorrhage	42	7.58%
GDM/ prediabetes	20	3.61%
Anemia	94	16.97%
Abnormal FHR pattern	32	5.78%
Malpresentation	77	13.90%
Oligohydramnios	44	7.94%
Polyhydramnios	10	1.81%
Medical illness (fever, UTI, gastroenteritis)	52	9.39%

**Table 4:** Management at admission and mode of delivery MgSO_4_: magnesium sulfate

Variables	Frequency (N=554)	Percentage (%)
Antenatal corticosteroids		
Complete course	389	70.22%
Incomplete course/none	165	29.78%
MgSO_4_ for neuroprotection	90	16.25%
Onset of labour		
Spontaneous	390	70.40%
Iatrogenic/medically indicated	164	29.60%
Mode of delivery		
Vaginal	331	59.75%
Instrumental vaginal	20	3.61%
Cesarean section	203	36.64%

The mean birth weight of preterm neonates in our study was 2205.76 ± 701.33 grams. Out of 1365 admissions to the NICU, 426 were preterm births. Thus preterm deliveries comprised 31.21% of all NICU admissions. Neonatal sepsis, jaundice requiring phototherapy, birth asphyxia, and RDS were the most frequent complications observed among preterm neonates requiring NICU admission (Table 5). Neonatal death occurred in 51 (9.21%) preterm infants with birth asphyxia being the most common cause accounting for 22 (43.14%) such deaths. The frequency of maternal factors, management, and adverse perinatal outcome variables was compared between the two groups (spontaneous and medically indicated preterm birth) and the significance of difference was derived (Table 6).

**Table 5:** Adverse perinatal outcome in preterm birth in the study NICU: neonatal intensive care unit; RDS: respiratory distress syndrome

Variables	Frequency (N=554)	Percentage (%)
Low birth weight	397	71.66%
NICU admission	426	76.90%
RDS	60	10.83%
Birth asphyxia	97	17.51%
Hypothermia	5	0.90%
Neonatal hypoglycemia	7	1.26%
Neonatal sepsis	109	19.68%
Jaundice requiring phototherapy	142	25.63%
Neonatal death	51	9.21%

**Table 6:** Comparison of maternal factors, management, and adverse perinatal outcome variables among spontaneous and medically indicated preterm birth groups PB: preterm birth; IUGR: intrauterine growth restriction, PROM: premature rupture of membranes; NICU: neonatal intensive care unit; RDS: respiratory distress syndrome

Variables	Spontaneous PB (N = 390) (%)	Iatrogenic/medically indicated PB (N=164) (%)	Chi-square	p-value
Prior preterm birth (N=63)	58 (14.87%)	5 (3.05%)	14.8607	0.000116
Preeclampsia/eclampsia (N=75)	15 (3.85%)	60 (36.59)	102.9417	<0.00001
IUGR (N=84)	14 (3.59%)	70 (42.68%)	134.1427	<0.00001
PROM (N=166)	125 (32.05%)	41 (25%)	2.7354	0.098149
Antepartum hemorrhage (N=42)	30 (7.69 %)	12 (7.32%)	0.0006	0.981265
Complete course of antenatal steroids (N= 389)	255 (65.38%)	134 (81.71%)	14.7085	0.000125
Cesarean section (N=203)	104 (26.67%)	99 (60.37%)	56.4747	<0.00001
Low birth weight (N=397)	269 (68.97%)	128 (78.05%)	4.6813	0.030493
NICU Admission (N=426)	299 (74.36%)	127 (77.44%)	0.0388	0.843916
RDS (N=60)	42 (10.77%)	18 (10.98%)	0.0061	0.937522
Birth asphyxia (N=97)	67 (17.18%)	30 (18.29%)	0.037	0.847519
Neonatal sepsis (N=109)	82 (21.03%)	27 (16.46%)	1.2455	0.264409
Jaundice requiring phototherapy (N=142)	98 (25.13%)	44 (26.83%)	0.0974	0.755
Mean duration of NICU stay (in days)	7.87 ± 1.98	8.42 ± 2.08	-	0.946963
Neonatal death (N=51)	36 (9.23%)	15 (9.15%)	0.0168	0.896899

## Discussion

Preterm birth is a syndrome with a heterogeneous etiology and underlying factors are usually divided into spontaneous and provider-initiated or medically indicated causes of preterm births. Complications of preterm birth are the single largest direct cause of neonatal deaths, responsible for 35% of such deaths annually, and the second most common cause of under-five deaths [6]. Addressing the global burden of preterm birth is critical to reducing neonatal and childhood mortality and to achieving the United Nations Sustainable Development Goal #3, to ensure healthy lives and promote well-being for all [10]. For a better understanding of the epidemiology of preterm birth and consequent improvement of quality of care to the mother and neonate, there is a need for detailed estimates of the burden of the condition, particularly in low- and middle-income countries where the data are scanty and not population-based [11].

The actual rate of preterm birth remains unexplained in many countries. It is estimated that 23.4% of global preterm births are reported from India making it the biggest contributor to preterm births worldwide [12]. The present study was retrospectively conducted in a tertiary care hospital in Eastern India over a period of one year. The incidence of preterm birth in our study population was 5.52%, which is similar to a study in Europe [13]. The preterm birth rate was 8.6% in an Indian multicentric study, which is similar to our study [10]. Another large longitudinal cohort study from India showed a higher frequency (14.9%) of preterm births [14]. The present study did not include pregnancies below 28 weeks of gestation, which may be the reason for the lower incidence.

Previous reports have indicated that extremes of maternal age (<20 years and >35 years) predispose to preterm birth [8,15]. This is in contrast to our study, which showed maximum prevalence (64.98%) in the 20-30 years age group. A study from Nepal showed similar age distribution as our study [16]. The risk of preterm births is seen to be higher among mothers with education lower than secondary level and belonging to lower socioeconomic classes [7,14,17]. The majority of our study population belonged to the rural community and lower socioeconomic class. However, more than 70% of these women were educated above the primary level. Our findings are in concordance with the study results from other Indian sites [10].

Gestational age at birth is the strongest predictor of neonatal complications and outcomes. The mean gestational age of preterm deliveries in our study was 34.39 ± 1.92 weeks. About 54.15% deliveries were in the 34-36^+6^ weeks group and rest were in the <34 weeks group. In an African study, the mean gestation was 33 ± 3 weeks and 62% were late preterms (34-36 weeks) [4]. The association between nulliparity and spontaneous preterm birth is supported by studies [18]. In the present study, about 47.29% women were nulliparous. A population-based study observed an increased risk for spontaneous preterm birth at < 37 weeks in nulliparous women and women in their fifth pregnancy compared to women in their second pregnancy [19]. There is conflicting evidence on the association of preterm birth with the number of ANC visits. While some studies showed a correlation between irregular ANC visits and preterm birth [17], a Belgian team found no substantial correlation between number of ANC visits and preterm birth but rather on the content and timing of care during pregnancy [20]. In our study, 32% of the women with preterm birth had irregular ANC.

Major factors associated with preterm birth include hypertensive disorders of pregnancy and preeclampsia, premature rupture of membranes, scarred uterus, IUGR, and placenta previa [21,22]. Factors associated with increased risk of preterm birth at India sites included severe antepartum haemorrhage, maternal hypertensive disorders, fetal malpresentation, and moderate/severe anaemia recorded anytime during pregnancy [10]. PROM was evident in 29.96% of our study participants; this finding accords with another Indian study from Kerela [23]. Anaemia, IUGR, and preeclampsia/eclampsia were the most common adverse pregnancy conditions prevalent in the present study after PROM. Most deliveries in our study were vaginal and about 36.64% were delivered by Cesarean section (CS). The prevalence of women with preterm birth delivered by CS ranged between 31% and 36.7% in the WHO Global and Multi-country Surveys [24]. The Department of Biotechnology (DBT) India Initiative study in North India showed a lower CS rate of 22.9% among preterm deliveries [14].

A preterm baby is prone to develop birth asphyxia due to an insufficient amount of surfactant in the lungs, which prevents atelectasis and maintains alveolar stability. The average birth weight of preterm babies tends to be significantly lower as compared to the term babies. The mean birth weight of preterm neonates in our study was 2205.76 ± 701.33 grams, which is similar to another Indian study in which the average preterm birth weight was 2.1 ± 0.6 kg [23]. The effect of preeclampsia, IUGR, and associated anaemia on the birth weight of premature babies has to be taken into consideration. About 76.9% of our preterm neonates required NICU admissions with neonatal sepsis (19.6%), jaundice (25.6%), birth asphyxia (17.5%), and respiratory distress syndrome (10.8%) being the most common complications. More than half (51.8%) of the preterm babies in Kerela required NICU admission [23]. From the literature review, it was found that all neonatal adverse outcomes including lower APGAR scores were significantly more frequent in preterm birth groups than in term groups [7,8,21]. Neonatal sepsis was encountered in 21.5% of admitted preterm neonates in an Egyptian study, which accords with our study [25]. APGAR scores predicted the risk of neonatal death among preterm infants across gestational-age strata. The relative risk of neonatal death consistently increased with decreasing APGAR scores in all gestational-age strata [26]. Neonatal death occurred in 51 (9.21%) preterm infants in our study with birth asphyxia being the commonest cause of such deaths.

Preterm birth has two major clinical etiologies: iatrogenic and spontaneous preterm birth. Iatrogenic preterm birth, including labour induction and CS delivery without labour, constitutes about 30-40% of all preterm births, and preeclampsia/eclampsia and severe intrauterine growth restriction are the common causes [7]. Spontaneous preterm birth can result from multiple causes, chiefly, infection or inflammation, cervical factors, haemorrhage, stress, genetics, and socio-demographic factors. Spontaneous preterm birth was noted in 70.40% of our study participants and the rest had induced/medically indicated preterm birth, a finding similar to the research studies from Africa [4,21].

Preeclampsia/eclampsia and IUGR were more significantly associated in the medically indicated group as compared to the spontaneous group. A Chinese study too revealed hypertensive disorder as the most common indication for early pregnancy termination in the iatrogenic group (72.8%), followed by fetal distress and placental abruption [27]. There was no significant difference in the frequency of antepartum haemorrhage between the spontaneous and iatrogenic groups in our study. In a study from Canada, the magnitude of association between placental abruption and the two subtypes of preterm birth was similar, whereas placenta previa was more strongly associated with iatrogenic preterm birth [28]. In the present study, a history of prior preterm birth was significantly associated with the spontaneous preterm birth group, a finding similar to the preterm Screening and Metabolomics in Brazil and Auckland (SAMBA) study [29]. The rates of CS were significantly higher in the iatrogenic preterm birth group (60.37%). This indicated that vaginal delivery was not preferred for most medically indicated preterm deliveries, which included hypertensive disorders and IUGR. Studies showed a higher risk of almost all adverse neonatal outcomes in medically indicated preterm birth compared to spontaneous preterm birth [29]. In contrast, we did not get any significant difference in the adverse neonatal outcomes between the two groups except for low birth weight, which was more frequent in the iatrogenic preterm birth group. More studies in the future can provide conclusive evidence regarding adverse perinatal outcomes in each subtype.

The limitations of our study included the retrospective study design. Also, the study did not include the subset of preterm births <28 weeks and lacked a control of term births for comparative analysis. However, we compared the two subtypes of preterm birth as the iatrogenic variety is on the rise due to recent advances in the management of high-risk factors. The first wave of the COVID-19 pandemic occurred during the last month of the study period, but it did not affect our research as none of the women or their neonates was found to be positive during the said period in our facility. Although larger multicentric prospective longitudinal studies can better define the association of maternal factors and perinatal outcomes with preterm birth, they are difficult to conduct in resource constraint settings. Further research is needed to analyze the long-term consequences on infant health and childhood neurodevelopment, explore the biochemical mechanism and genetics of preterm birth, and study the effects of screening and preventive strategies.

## Conclusions

Preterm birth causes a remarkable increase in adverse perinatal outcomes in terms of neonatal morbidity and mortality as compared to term birth. The burden of preterm birth on the health system of the developing world is particularly substantial. A regular goal-oriented population-based clinical audit into sociodemographic profile, risk factors, and perinatal morbidity and mortality associated with this condition might improve the clinical outcomes by unravelling the pathogenesis specific to that population. The findings of this study provide a general overview of the associated etiological factors and perinatal health concerns associated with preterm birth in a rural cum semi-urban setting of Eastern India and the influence of management in a tertiary hospital. Our study might provide health care providers and policymakers with essential data for taking steps toward the prevention and management of preterm birth from a developing country’s perspective.
